# Caught in the thickness of brain fog: exploring the cognitive symptoms of Chronic Fatigue Syndrome

**DOI:** 10.3389/fphys.2013.00063

**Published:** 2013-04-05

**Authors:** Anthony J. Ocon

**Affiliations:** Departments of Physiology/Medicine, Center for Hypotension, New York Medical CollegeValhalla, NY, USA

**Keywords:** Chronic Fatigue Syndrome, postural orthostatic tachycardia syndrome, neurocognition, cerebral blood flow (CBF), functional magnetic resonance imaging (fMRI), brain fog, orthostatic intolerance

## Abstract

Chronic Fatigue Syndrome (CFS) is defined as greater than 6 months of persistent fatigue that is experienced physically and cognitively. The cognitive symptoms are generally thought to be a mild cognitive impairment, but individuals with CFS subjectively describe them as “brain fog.” The impairment is not fully understood and often is described as slow thinking, difficulty focusing, confusion, lack of concentration, forgetfulness, or a haziness in thought processes. Causes of “brain fog” and mild cognitive impairment have been investigated. Possible physiological correlates may be due to the effects of chronic orthostatic intolerance (OI) in the form of the Postural Tachycardia Syndrome (POTS) and decreases in cerebral blood flow (CBF). In addition, fMRI studies suggest that individuals with CFS may require increased cortical and subcortical brain activation to complete difficult mental tasks. Furthermore, neurocognitive testing in CFS has demonstrated deficits in speed and efficiency of information processing, attention, concentration, and working memory. The cognitive impairments are then perceived as an exaggerated mental fatigue. As a whole, this is experienced by those with CFS as “brain fog” and may be viewed as the interaction of physiological, cognitive, and perceptual factors. Thus, the cognitive symptoms of CFS may be due to altered CBF activation and regulation that are exacerbated by a stressor, such as orthostasis or a difficult mental task, resulting in the decreased ability to readily process information, which is then perceived as fatiguing and experienced as “brain fog.” Future research looks to further explore these interactions, how they produce cognitive impairments, and explain the perception of “brain fog” from a mechanistic standpoint.

## Introduction

**Figure 1:**
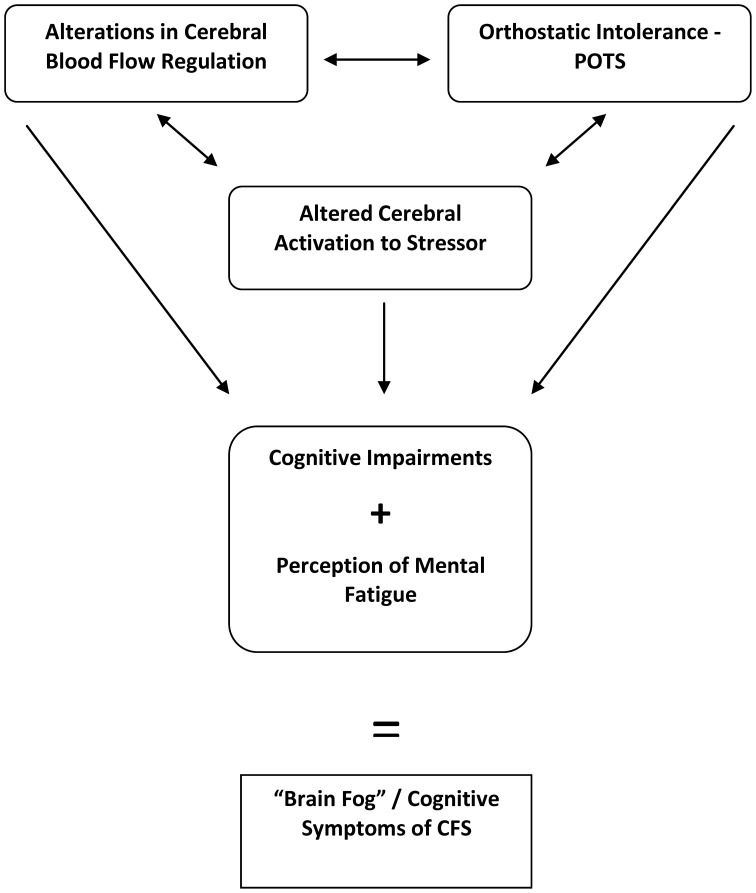
**Interactions between multiple factors may contribute to the cognitive symptoms subjectively described as “brain fog” in Chronic Fatigue Syndrome (CFS).** POTS, Postural Tachycardia Syndrome.

Chronic Fatigue Syndrome (CFS) is a clinically defined set of symptoms of unknown etiology most notable for persistent fatigue lasting greater than 6 months. In 1994, the Center for Disease Control and Prevention (CDC) uniformly defined CFS. The CDC requiring the fatigue to be of new onset, non-exertional, not improved with rest, and debilitating to a person's lifestyle (Fukuda et al., 1994). Additionally, at least four of the following symptoms must be concurrently present: pharyngeal pain, cervical or axillary lymphadenopathy, myalgia, polyarthritis without erythema or edema, headache, non-restful sleep, prolonged post-exercise fatigue, and/or debilitating cognitive impairments in short-term memory and concentration (Fukuda et al., 1994). While the exact symptoms of each case of CFS are heterogeneous, up to 85% of individuals describe experiencing cognitive impairments (Komaroff, 1993). These cognitive impairments have subjectively been described by patients with CFS as “brain fog.” Descriptions of “brain fog” include slow thinking, difficulty focusing, confusion, lack of concentration, forgetfulness, or a haziness in thought processes. In fact, “brain fog” may be one of the most debilitating aspects of CFS (Jain and DeLisa, 1998; Natelson and Lange, 2002; Afari and Buchwald, 2003; Jorgensen, 2008). However, a precise definition of CFS “brain fog” has yet to be formulated. Thus, “brain fog” may be conceptionally defined as the perception and experience of mental fatigue that is associated with and related to mild cognitive impairments in CFS. Research has focused on describing “brain fog” and mild cognitive impairment in regards to CFS using objective measurements. Physiologically, areas of study have investigated mental fatigue and impairment as the effects of orthostatic stress, in relationship to changes in cerebral blood flow (CBF), and as a perception. In addition, neurocognitive testing has localized the cognitive impairments in CFS to the domains of attention, information processing, memory, and reaction time (Cockshell and Mathias, 2010). Furthermore, functional magnetic resonance imaging (fMRI) of the brain has associated changes in anatomical structures to the cognitive fatigue experienced in CFS. However, while much research has been done to analyze the individual components of the cognitive symptoms in CFS, no single source has compiled a comprehensive description of the multiple factors and their interactions that may play a role in the CFS patient's experience of “brain fog” (see Figure 1). Thus, to better understand cognitive impairment and “brain fog” in CFS, as well as to guide future research, the goal of this review is to summarize, standardize, and analyze the cognitive symptoms which lead to impairment.

## Neurocognitive testing demonstrates specific cognitive deficits in CFS

If “brain fog” truly is the subjective experience of cognitive impairment in CFS, the impairment should be measurable. Early work using sensory and cognitive event-related potentials broadly demonstrated deficits in CFS subjects compared to control subjects in areas of memory, concentration, attention, information processing, and reaction time (Prasher et al., 1990). Research has attempted to use more focused tests to narrow in on what exactly these deficits are. A table of cognitive tests that have been used to study impairments in CFS is listed (see Table 1).

**Table 1:** **List of cognitive tests used to study Chronic Fatigue Syndrome cognitive impairments**.

**Name of test used**	**References**
Paced auditory serial addition test	DeLuca et al., 1993, 1995
Digit span test	DeLuca et al., 1993, 1995
Trail making test	DeLuca et al., 1995
Booklet category test	DeLuca et al., 1995
Rey complex figure test	DeLuca et al., 1995
California verbal test	DeLuca et al., 1995
Wechsler adult intelligence scale revised	DeLuca et al., 1995
Logical memory subtest of the Wechsler memory scale revised	DeLuca et al., 1995
Beck depression inventory	Grafman et al., 1993; DeLuca et al., 1995
Wechsler adult intelligence scale revised	Grafman et al., 1993
Simple reaction time test	Grafman et al., 1993
Serial reaction time test	Grafman et al., 1993
Time wall test	Grafman et al., 1993
Time clock test	Grafman et al., 1993
Tower of London	Grafman et al., 1993
Tower of Hanoi	Grafman et al., 1993
Twenty questions test	Grafman et al., 1993
Wechsler memory scale revised	Grafman et al., 1993
Experimental paired associate test	Grafman et al., 1993
Hasher frequency monitoring task	Grafman et al., 1993
Story memory test	Grafman et al., 1993
Word fluency test	Grafman et al., 1993
Somatization scale	Grafman et al., 1993
Neurobehavioral rating scale	Grafman et al., 1993
Fatigue scale	Grafman et al., 1993
Cambridge neuropsychological test automated battery	Joyce et al., 1996; Capuron et al., 2006
Digit span forward test	Dobbs et al., 2001
Digit span backward test	Dobbs et al., 2001
Trails A test	Dobbs et al., 2001
Trails B test	Dobbs et al., 2001
*n*-back test	Caseras et al., 2006; Ocon et al., 2012; Stewart et al., 2012
Finger tapping task	Cook et al., 2007
Auditory monitoring task	Cook et al., 2007
Motor imagery task	de Lange et al., 2004
Control visual imagery task	de Lange et al., 2004

Using the Paced Auditory Serial Addition Test (PASAT) and the Digit Span Test, tests of memory, attention, and data processing, DeLuca et al. (1993) found that CFS subjects scored lower than control subjects, suggesting deficits in information processing of complex material. Further work by DeLuca et al. (1995) showed that CFS subjects have intact memory storage, consolidation, and retrieval and “higher order” cognitive function; however, they exhibit decreased speed and efficiency in information processing, especially with auditory material, and subjectively perceive a more generalized cognitive impairment (DeLuca et al., 1995). Interestingly, this perception of generalized cognitive impairment fits the concept of “brain fog.”

In addition to information processing, deficits in memory have been studied in CFS. Work by Grafman et al. (1993) compared responses to a battery of memory and neuropsychological tests (see Table 1) between CFS and control subjects. While overall memory consolidation and storage were intact, CFS subjects demonstrated selective deficits in memory processing (Grafman et al., 1993). Joyce et al. (1996) used the Cambridge neuropsychological test automated battery (CANTAB), which is a set of tests that measure various aspects of memory, attention, reaction time, executive functioning, and decision making, and found that CFS subjects exhibited deficits in working memory and impaired attention. Working memory may be defined as a transient cognitive storage system that is able to process information and use it to complete a task (Baddeley, 1986). Similarly, Dobbs et al. (2001) reported that CFS subjects had working memory deficits based on results from the Digit Span Forward test, Digit Span Backward test, Trails A test, and Trails B test, which are a set of memory and attention tests. These deficits were mainly apparent during challenging tasks that needed sustained attention and efficient mental processing (Dobbs et al., 2001). Work by Capuron et al. (2006), using the CANTAB, further distinguished that working memory and impaired attention were only impaired compared to control subjects in a subgroup of CFS patients who subjectively stated experiencing increased mental fatigue. When they compared CFS who did not report mental fatigue, they did not find a difference (Capuron et al., 2006).

Of note, children with CFS have also been studied and have been shown to exhibit cognitive impairment in the areas of focusing, splitting, switching attention, working memory, and auditory learning (Haig-Ferguson et al., 2009). Interestingly, a recent study in children with CFS found that treatment with both cognitive behavioral therapy and pharmacotherapy lead to improvements in attention (Kawatani et al., 2011).

In summary, research has demonstrated cognitive impairment in CFS, particularly in working memory, information processing, attention, and reaction time. Deficiencies in these cognitive areas may be perceived on a day-to-day basis as “brain fog.” Mental fatigue is necessary and must be experienced in order for CFS subjects to have cognitive symptoms. Comprehensively, the cognitive impairments associated with CFS impair the ability to maintain attention for an extended period of time, disrupt efficient and speedy information processing, and results in an inability to plan or order responses from memory (Joyce et al., 1996). Functionally, this is translated into increased reaction time while completing tasks. The deficits in information processing and working memory are similar and most likely related. Task difficulty plays a role in the perception of mental fatigue, with CFS subjects feeling most fatigued following the more difficult tasks. Additionally, the greatest degree of impairment and disability has been noted in CFS subjects who perform the worst on neurocognitive tests (Christodoulou et al., 1998).

## Psychiatric comorbidities are not associated with cognitive impairment in CFS

Depression and anxiety often co-exist in subjects with CFS and may in themselves be associated with cognitive impairment (Afari and Buchwald, 2003). Studies have focused on determining whether comorbid psychiatric illness is the cause of cognitive impairment in CFS.

An early study prior to the 1994 CDC definition suggested that subjective cognitive impairment in individuals with CFS may be associated with depression (McDonald et al., 1993). However, a study by DeLuca et al. (1997) suggested otherwise. This group separated CFS subjects into a cohort with psychiatric comorbidity and a cohort without. When compared to healthy control subjects' performance on neurocognitive tests, CFS subjects without psychiatric comorbidities exhibit neurocognitive impairment, whereas those with comorbidities did not (DeLuca et al., 1997). More recent work by the same group similarly concluded that CFS subjects without psychiatric comorbidities exhibit deficits in working memory and information processing, while those with psychiatric comorbidities were not different from controls (DeLuca et al., 2004). Further work by other groups similarly has concluded that the cognitive impairment in CFS is not due to depression (Constant et al., 2011; Santamarina-Perez et al., 2011).

From these studies, two distinct groups of CFS subjects have been identified. It appears that cognitive symptoms occur only in CFS subjects without psychiatric comorbidities. More studies are needed to elucidate the factors which may explain this. There is a clear need in future studies to appropriately stratify CFS subjects into those with and without psychiatric comorbidities.

## Orthostatic stress impairs cognitive performance in CFS

Cognitive impairment has been associated with the physiological onset of orthostatic intolerance (OI). OI is defined by the onset of signs and symptoms that occur when an individual assumes the upright posture, with the signs and symptoms being alleviated by resuming the supine position. Rowe et al. (1995) first described OI in CFS in 1995. Both adults and adolescents with CFS often experience OI in the form of the Postural Tachycardia Syndrome (POTS), with syncope being less common (De et al., 1996; Rowe and Calkins, 1998; Stewart et al., 1999; Karas et al., 2000; Hoad et al., 2008). POTS is defined in adults as symptomatic OI with an increase in HR of at least 30 beats per minute (bpm) or a maximum HR of 120 bpm (Schondorf and Low, 1993). Typical symptoms of OI that occur include dizziness, neurocognitive impairment, tremulousness, nausea, and long-term fatigue (Schondorf and Freeman, 1999; Medow and Stewart, 2007). Some individuals with POTS experience migraine headaches (Piovesan et al., 2008). While standing upright, physiologic changes noted in POTS include increased diastolic BP and total peripheral resistance, decreased stroke volume, and impaired venoconstriction (Low et al., 1994). Regional venous blood pooling may occur while upright, particularly in the splanchnic region and in the legs (Stewart and Montgomery, 2004). POTS subjects tend to have decreased total blood volumes, red cell volume, and plasma aldosterone (Raj et al., 2005). About half of the individuals with POTS exhibit orthostatic hyperventilation and hypocapnia, while the other half breathe normally and are eucapnic (Stewart et al., 2006). A similar respiratory pattern of orthostatic hypocapnia has been noted in about a third of CFS subjects (Naschitz et al., 2000, 2006; Natelson et al., 2007).

Limited psychological testing has occurred in POTS subjects. Work by Raj et al. (2009) found mild depression and moderate anxiety to be prevalent in POTS subjects, but lifetime prevalence of psychiatric diseases overall was not different than that of control subjects. They determined that the anxiety experienced in POTS was more related to the symptoms of the disease and how those symptoms may affect the individual (Raj et al., 2009). Similar to CFS, POTS subjects may also experience attention deficits (Raj et al., 2009).

Moreover, some of the symptoms of CFS and POTS overlap in their clinical descriptions as well as in their associations with cognitive impairment, and individuals commonly present with symptoms of both syndromes (De et al., 1996; Hoad et al., 2008). Of note, some individuals with CFS and/or POTS also suffer from Ehlers–Danlos Syndrome, a genetic disorder that causes a defect in the synthesis of collagen which affects connective tissue (Rowe et al., 1999; Mathias et al., 2012). This syndrome may predispose to OI due to the occurrence of excessive venous pooling because of exaggerated venous wall distention and dysfunctional venous valves (Rowe et al., 1999).

Neurocognitive impairment is often described as a symptom of OI. The subjective descriptions of the cognitive impairments in POTS are similar to those in CFS. Therefore, OI, in particular POTS, may be connected to the “brain fog” experienced in CFS. Limited work has looked at neurocognitive testing during orthostatic stress. One study by Karas et al. (2000) found that adolescents with POTS experience cognitive impairment, although no formal neurocognitive testing was used to determine the exact areas that were affected. Using more objective measures, work by Ocon et al. (2012) studied subjects with both CFS and POTS (CFS/POTS) during graded orthostatic stress, using a *n*-back task as a cognitive stressor. The *n*-back task is a cognitive test of progressively increasing difficulty that stresses domains of working memory and information processing while measuring reaction time (Braver et al., 1997). Results showed that subject with CFS/POTS exhibited no differences in accuracy or reaction time compared to control subjects while supine (Ocon et al., 2012). However, during moderate to severe levels of orthostatic stress, CFS/POTS subjects were less accurate and had a longer reaction time compared to control subjects, particularly during the difficult stages of the *n*-back task (Ocon et al., 2012). This study demonstrated that orthostatic stress may impair the cognitive abilities of those with CFS/POTS, especially during difficult tasks. Thus, working memory, information processing, and reaction time may be impaired with prolonged orthostatic stress. These cognitive deficiencies may be perceived as “brain fog.” Speculatively, one may consider physiological changes during orthostasis to potentially be a cause of the cognitive impairment. In particular, alterations in CBF regulation may be related to the cognitive symptoms as described below.

## Cerebral blood flow is decreased in CFS

Much research has studied CBF alterations in CFS. Early work measured regional CBF using single photon emission computed tomography (SPECT) in those with CFS. Ichise et al. (1992) found that 80% of CFS subjects exhibited decreased regional CBF particularly in the frontal, temporal, parietal, occipital, and basal ganglia regions compared to control subjects without CFS or other neurological/neuropsychiatric disorders. Similarly using SPECT, Schwartz et al. (1994) found decreased regional CBF in CFS subjects in the frontal and temporal lobes. Costa et al. (1995) studied brainstem perfusion in CFS subjects and found hypoperfusion compared to both control subjects and subjects with major depression. However, work by Fischler et al. (1996) did not find significant differences in regional CBF between CFS and control subjects, and MacHale et al. (2000) actually found increased CBF in CFS subjects in the thalamus, pallidum, and putamen regions. Technical differences with SPECT and methodological inconsistencies between studies may account for the contradictory results.

Other techniques of measuring CBF have been applied to determine whether decreased CBF does occur in CFS. Positron emission tomography was used by Tirelli et al. (1998) to look at cerebral metabolism in CFS subjects. They found that CFS subjects exhibited hypometabolism in the right mediofrontal cortex and brainstem compared to control subjects (Tirelli et al., 1998). Near-infrared spectroscopy (NIRS) is another method which looks at changes in brain oxygenation of hemoglobin. NIRS measurements by Tanaka et al. (2002) found that CFS subjects had lower brain oxy-hemoglobin while standing upright compared to control subjects. This suggests that orthostatic stress may negatively influence cerebral oxygenation in CFS. Similarly, Patrick et al. (2008) used NIRS during maximal incremental cycle exercise in female CFS subjects. They found that the CFS subjects exhibited exercise intolerance that was associated with decreased prefrontal oxygenation (Patrick et al., 2008). Additionally, Natelson et al. used more advanced imaging techniques of Xenon gas diffusion computerized tomography (Yoshiuchi et al., 2006) and magnetic resonance arterial spin labeling (Biswal et al., 2011) to demonstrate reduced total CBF in CFS subjects compared to controls, especially in CFS subjects without psychiatric comorbidities.

In order to study whether altered CBF affected cognitive performance, Schmaling et al. (2003) used SPECT measurements of CBF during the PASAT test to see whether impaired CBF in CFS could be associated with decreased cognitive impairment. Prior to testing, CFS subjects had decreased perfusion in the anterior cingulate region of the brain compared to control subjects (Schmaling et al., 2003). During testing, there was a greater increase in blood flow to the left anterior cingulate region in CFS subjects (Schmaling et al., 2003). Furthermore, CFS subjects exhibited a wide-spread, diffuse pattern of CBF in the frontal lobe, temporal lobe, and thalamus compared to a small, focal pattern in the right frontal lobe and right temporal lobe in control subjects (Schmaling et al., 2003). No differences in test performance occurred between groups, but CFS subjects reported greater mental fatigue following testing. This suggested that an increased change in cerebral perfusion with greater activation of more brain regions was necessary for CFS subjects to perform at the same level as that of control subjects (Schmaling et al., 2003). This may be due to the need to overcome the decreased baseline CBF. These subjects also subjectively experienced “brain fog” as mental fatigue, suggesting that the increased change in cerebral perfusion and activation during cognitive tasks was perceived as stressful and exaggerated exhaustion. Speculatively, this may be related to altered cerebral metabolism, with increased production of metabolites affecting neuronal biochemical pathways. Further research measuring cerebral metabolism during neurocognitive testing is needed to detail the processes of mental fatigue and impairment in CFS.

As mentioned above, orthostatic stress is related to neurocognitive impairments in CFS and POTS and may induce changes in CBF. Since some POTS symptoms overlap with CFS, it is important to look at how CBF is affected in this syndrome. In most studies, CBF was estimated by measurements of CBF velocity (CBFv) using transcranial Doppler sonography. Work by Novak et al. (1998) found that during orthostatic stress, POTS subjects have decreased CBFv. This may be related to hyperventilation and hypocapnia-induced increased cerebrovascular resistance (Novak et al., 1998). Similarly, Low et al. (1999) suggested that the altered CBFv in POTS was explained solely by respiratory changes and their concomitant affect on cerebrovasomotor tone. Other research suggested that ineffective cerebral autoregulation also played a role in the decreased CBFv seen in POTS (Jacob et al., 1999; Hermosillo et al., 2002). Ocon et al. (2009) found a relationship between decreased CBFv and altered cerebral autoregulation in a cohort of POTS subjects who were eucapnic during orthostatic stress. However, Schondorf et al. (2005) reported normal cerebral autoregulation in POTS subjects, though the group did not measure changes in CO_2_. Thus, CBF appears to be decreased in POTS subjects similarly to how it is decreased in CFS subjects, but the mechanisms involved are controversial and probably multifactorial. The decreased CBF in POTS and CFS during orthostatic stress may play a role in their perceived cognitive impairment.

To study this, work by Stewart et al. (2012) looked at how CBFv changed during increasing mental and orthostatic stress in a group of subjects with both CFS and POTS. Mental stress was induced by the *n*-back task, while orthostatic stress was induced with graded tilt-table testing. In control subjects, CBFv increased as the level of *n*-back difficulty increased, independent of orthostatic stress (Stewart et al., 2012). In contrast, CFS/POTS subjects did not exhibit an increase in CBFv with increasing *n*-back difficulty. Additionally, decreasing CBFv in CFS/POTS subjects was dependent on the level of orthostatic stress (Stewart et al., 2012). This study concluded that control subjects demonstrated appropriate neurovascular coupling to mental stress, whereas CBFv is not activated by mental stress in CFS/POTS subjects (Stewart et al., 2012). This may be due to an uncoupling of cognitive–vasomotor interactions (Stewart et al., 2012). Additionally, superimposed orthostatic stress appears to further negatively impact the cerebral vasomotor responses to mental stress in CFS/POTS subjects (Stewart et al., 2012).

Therefore, cognitive impairments in CFS/POTS may be related to impaired activation and regulation of CBF, especially during challenging mental tasks and orthostatic stress. Adequate cerebral perfusion is necessary for the brain to function. In those with CFS and/or POTS, a gradual and chronic hypoperfusion of the brain may occur, especially during orthostatic stress (Ocon et al., 2009). Animal studies suggest that chronic cerebral hypoperfusion may cause cognitive impairment (Ni et al., 1994; Liu et al., 2012). Thus, if CBF is decreased chronically in CFS, mild cognitive impairment may be the result.

## Functional MRI finds altered regional cerebral activation in CFS

Cerebral imaging techniques have tried to associate cognitive impairments with specific cerebral regions. An early MRI study by Lange et al. (1999) found increased hyperintensities in the frontal lobe subcortical white matter of CFS subjects without psychiatric comorbidities compared to those with comorbidities and control subjects, suggesting nonspecific cerebral lesions as the basis for cognitive impairment. Using fMRI to measure cerebral changes during cognitive tasks (see Table 1), de Lange et al. (2004) found that CFS subjects exhibited decreased caudate nucleus activity, increased recruitment of cerebral regions, and an inactive ventral anterior cingulated cortex during errors compared to control subjects. They suggested that these changes may be associated with fatigue, the need for additional neural activation to complete a task, and reduced motivation (de Lange et al., 2004). Additional work using fMRI found that demanding working memory tasks elicited activation in more regions of the brain in CFS subjects than in control subjects (Lange et al., 2005). It is tempting to speculate that CFS subjects may require more brain activation during working memory tasks. Consistent with this speculation, Caseras et al. (2006) used the *n*-back task to monitor how CFS subjects' brains respond to different levels of mental stress compared to control subjects. They found that during lower difficulty *n*-back testing, CFS patients exhibited increased activation of working memory centers (Caseras et al., 2006). However, during higher difficulty testing, they had decreased activation compared to control subjects (Caseras et al., 2006). Similarly, Cook et al. (2007) showed that CFS subjects, when compared to control subjects, have increased cortical and subcortical cerebral activation during fatiguing, stressful mental tasks (see Table 1). They suggested that increased brain activation may be related to the perception of mental fatigue (Cook et al., 2007).

## Physical and cognitive symptoms of CFS may be a mental perception of stimuli

Prior to the 1994 CDC definition of CFS, Kent-Braun et al. (1993) theorized that the physical symptoms of fatigue in CFS were in part related to a central nervous system perception of the body's response to a stressor. They hypothesized that CFS did not induce true muscle fatigue, but rather the experience of fatigue was a mental discernment of peripheral sensations and stimuli. To test this hypothesis, the group studied muscle fatigue in the tibialis anterior muscle following exercise. They found that CFS subjects failed to voluntarily activate the muscle after prolonged stress, despite no detectable metabolic or functional changes having occurred (Kent-Braun et al., 1993). This suggested that in CFS bodily fatigue during and following exercise was perceived mentally rather than there being true peripheral muscle exhaustion. Another study further examined fatigue as a perception. While studying post-exertion fatigue in females with CFS, Sisto et al. (1998) found that the experience of fatigue following exercise was exaggerated compared to control subjects, and it may experienced in a prolonged manner for 12–14 days. Together, these studies suggest that the perception of fatigue in CFS is a mental experience of physical fatigue following exercise, or potentially any a stressful stimulus, even if no such physical exhaustion occurs. In addition to the cognitive component, this mental experience is part of the subjective “brain fog” that patients with CFS describe. This perception may be exaggerated, continual, and prolonged, despite true muscle exhaustion not occurring.

In an attempt to detail a mechanism describing the perception of fatigue in CFS, recent work has looked at neuroactive amino acids and metabolite concentrations following exercise. A study found increased tryptophan, decreased branch chain amino acids, and decreased tyrosine following exercise in the plasma of CFS subjects compared to controls (Georgiades et al., 2003). The exact meaning of these changes is difficult to discern, but speculatively this may suggest that metabolic alterations in precurors involved in the serotonergic and dopaminergic pathways may be involved in the central perception of fatigue. Moreover, another study showed that CFS subjects exhibit electroencephalogram changes during fatiguing exercise. Again, how this relates to the perception of fatigue is speculative, but it may suggest that altered cerebral signaling, particularly that involved with motor activity, may be experienced as mental fatigue (Siemionow et al., 2004). Further work is needed to determine how mental fatigue, cognitive impairment, and the subjective experience of “brain fog” relate biochemically, physiologically, and mechanistically to the changes that occur in CFS following exercise or any stressor.

## The experience of “brain fog” in CFS as a collection of physiological, cognitive, and perceptual factors

Over the past 20 years, research has answered many questions regarding cognitive symptoms in CFS. The experience of “brain fog” in those with CFS appears to be the conscious perception of cognitive impairment and is related to mental fatigue. Individuals with CFS do not exhibit complete cognitive disability or dementia. However, they may experience deficits in working memory, information processing, and attention, which then may translate into a longer reaction time during tasks. It is logical that the perception of such deficits would be described as mental cloudiness and difficulty thinking.

Drawing from an overview of the above studies, cognitive impairment in CFS is exacerbated by stressful stimuli such as a difficult mental task, exercise, and/or orthostatic stress. Physiologically, the cognitive impairments may be associated with impaired cardiovascular hemodynamics as seen in POTS, decreased total CBF, and altered activation of CBF during mental tasks. Psychiatric diseases do not seem to be related to cognitive impairment. Importantly, the perception of the symptoms of CFS is often exaggerated and excessive compared to what is measured. Aside from the cognitive impairments noted above, this perception may be disabling. Thus, “brain fog” is caused by measurable cognitive impairments but also the more subjective perception of mental fatigue and its impact on the individual with CFS. As shown in Figure 1, interactions of multiple factors clearly play a role. Based on the above research, one speculative view of the mechanisms behind the cognitive symptoms of CFS is that altered CBF activation and regulation are exacerbated by a stressor, such as orthostasis or a difficult mental task, that results in the decreased ability to readily process information, which is then perceived as fatiguing and experienced as “brain fog.” Thus, future research will study if interventions that modify CBF affect the cognitive symptoms of CFS. Additionally, more work will look at how orthostatic stress impacts neurocognition. Finally, since the impaired cognitive domains in CFS have been determined, interventions to improve these domains will be tested.

To date, treatment of CFS has been focused on improving the physical fatigued state rather than the cognitive impairment. Meta-analyses demonstrate that cognitive behavioral therapy and graded exercise therapy effectively treat CFS in many individuals (Edmonds et al., 2004; Price et al., 2008). It would not be surprising if mental fatigue also is improved with such therapies, but future studies are necessary to formally determine this.

### Conflict of interest statement

The author declares that the research was conducted in the absence of any commercial or financial relationships that could be construed as a potential conflict of interest.
